# Akirin2 regulates proliferation and differentiation of porcine skeletal muscle satellite cells via ERK1/2 and NFATc1 signaling pathways

**DOI:** 10.1038/srep45156

**Published:** 2017-03-22

**Authors:** Xiaoling Chen, Yanliu Luo, Zhiqing Huang, Gang Jia, Guangmang Liu, Hua Zhao

**Affiliations:** 1 Key Laboratory for Animal Disease-Resistance Nutrition of China Ministry of Education, Institute of Animal Nutrition, Sichuan Agricultural University, Chengdu, Sichuan 611130, P. R. China

## Abstract

Akirin2, a novel nuclear factor, plays an important role in myogenesis. To investigate the role of Akirin2 in proliferation and differentiation of porcine skeletal muscle satellite cells, Akirin2 overexpression and Akirin2 silence technologies were employed. Our results showed that overexpression of Akirin2 markedly enhanced the proliferation and differentiation of porcine skeletal muscle satellite cells, whereas silencing of Akirin2 got the opposite results. Furthermore, our results showed that Akirin2 affected proliferation and differentiation of porcine skeletal muscle satellite cells through extracellular-signal regulated kinase-1/2 (ERK1/2) and NFATc1 signaling pathways. These results indicate that Akirin2 can effectively promote skeletal muscle satellite cells proliferation and differentiation, acting through ERK1/2- and NFATc1-dependent mechanisms.

Skeletal muscle is mainly composed of postmitotic multinucleated muscle fibers (myofibers). Myogenesis is a multistep process, which involves skeletal muscle satellite cell activation, proliferation, differentiation and formation of multinucleated myofibers^1^,^2^. The entire process is regulated by large number of extracellular factors and some distinct signaling pathways, resulting in the subsequent activation of major transcription factors in regulating gene expression^3^,^4^.

The extracellular signal-regulated kinase 1/2 (ERK1/2) pathway, a part of the mitogen-activated protein kinase (MAPK) pathway, is involved in regulating many facets of cellular processes such as cell proliferation, differentiation and death^5^. Previous studies showed that ERK1/2 is required for myoblast proliferation and differentiation^6^,^7^,^8^,^9^. The activated ERK1/2 pathway enhances the skeletal muscle cell proliferation, but negatively regulates myoblast differentiation^10^,^11^. There is some evidence that nuclear factor of activated T cells c1 (NFATc1) cooperates with ERK1/2 signaling in the induction of cell proliferation, apoptosis and differentiation^12^,^13^,^14^.

The nuclear factor Akirin2, a member of the Akirin family, plays a fundamental role in myogenesis^15^. It may be considered as a potential functional candidate gene of meat quality^16^,^17^. The ERK1/2 signaling pathway has been shown to be regulated by Akirin2 in tumor cell lines^18^,^19^. However, whether and how Akirin2 affects skeletal muscle cell proliferation and differentiation remains unclear.

In our previous studies, we cloned porcine Akirin2 and examined its effects on expressions of IL-6 and myosin heavy chain (MHC) isoform^17^,^20^. In the present study, Akirin2 overexpression and Akirin2 silence were employed to study the effects and the underlying mechanism of Akirin2 on the proliferation and differentiation of porcine skeletal muscle satellite cells.

## Results

### Expression of Akirin2 in proliferating and differentiating porcine skeletal muscle satellite cells

**Figure 1:**
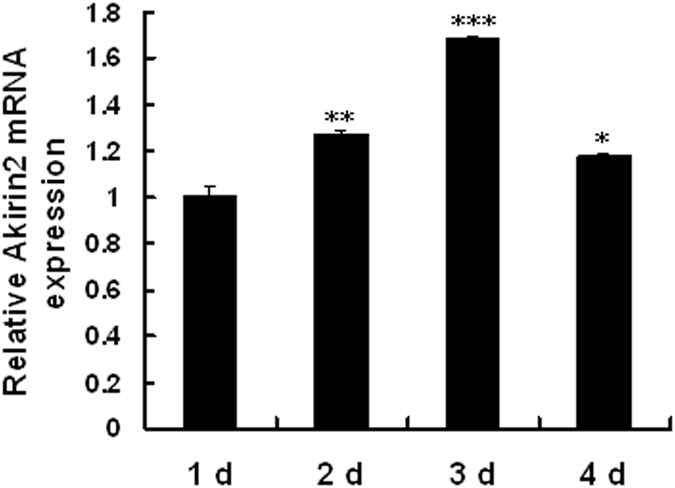
Relative expression of *Akirin2* mRNA during porcine skeletal muscle satellite cells proliferation. RNA was extracted from the proliferating porcine skeletal muscle satellite cells on the days 1, 2, 3, and 4. *Akirin2* mRNA expression was analyzed by real-time quantitative PCR. The amount of *Akirin2* mRNA was normalized to the amount of *GAPDH* mRNA. Data were presented as means ± SE (n = 3). *P < 0.05, **P < 0.01 and ***P < 0.001 as compared with the control group (1 d).

**Figure 2:**
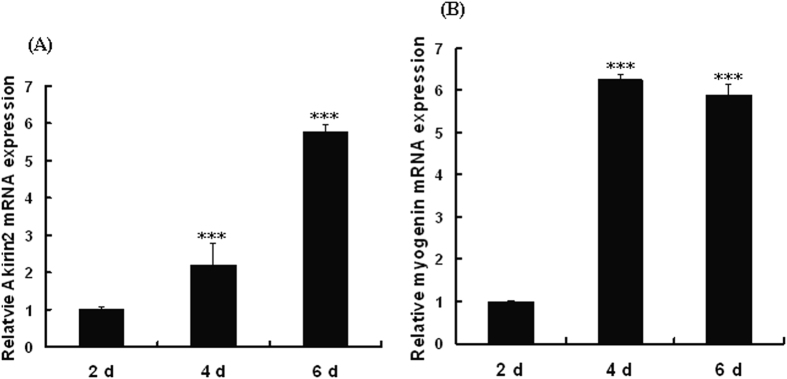
Relative expression of *Akirin2* mRNA during porcine skeletal muscle satellite cells differentiation. RNA was extracted from the differentiating porcine skeletal muscle satellite cells on the days 2, 4, and 6. *Akirin2* (**A**) and *myogenin* (**B**) mRNA expression was analyzed by real-time quantitative PCR. The amount of *Akirin2* and *myogenin* mRNA was normalized to the amount of *GAPDH* mRNA. Data were presented as means ± SE (n = 3). ***P < 0.001 as compared with the control group (2 d).

Akirin2 expression in proliferating and differentiating porcine skeletal muscle satellite cells was assessed by real-time quantitative PCR. As shown in Fig. 1, *Akirin2* mRNA was was upregulated during porcine skeletal muscle satellite cells proliferation. We also found that *Akirin2* mRNA was upregulated during porcine skeletal muscle satellite cells differentiation, which was similar to *myogenin* mRNA (Fig. 2).

### Akirin2 promotes proliferation of porcine skeletal muscle satellite cells

**Figure 3:**
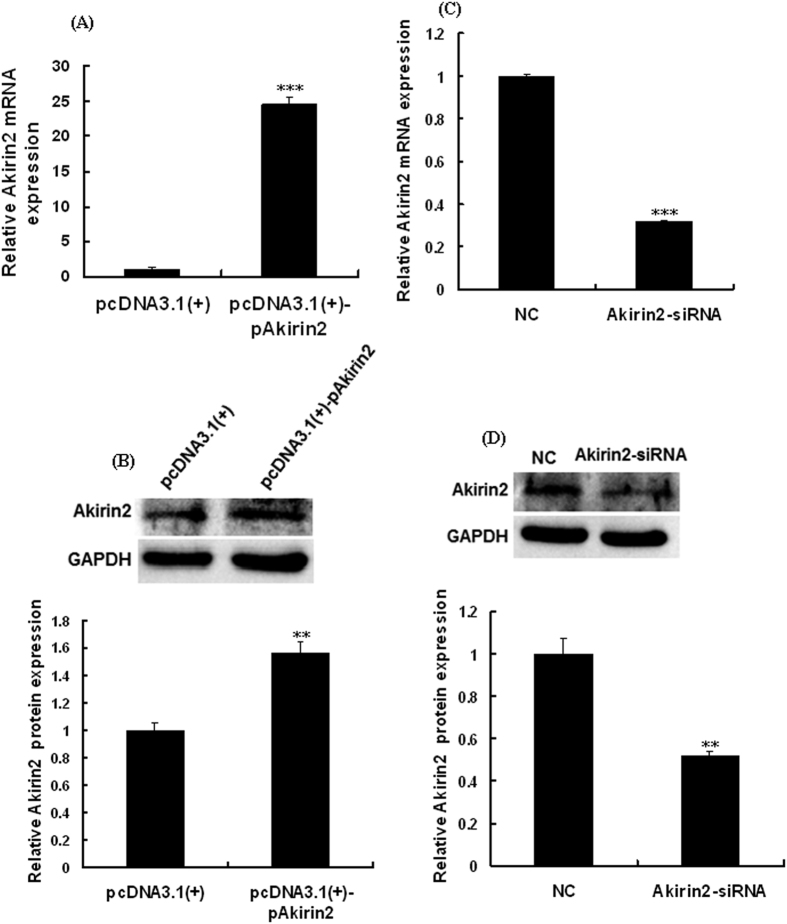
Effect of Akirin2 overexpression and Akirin2 silencing on the mRNA and protein expression levels of Akirin2 in porcine skeletal muscle satellite cells. Approximately 60% confluent porcine skeletal muscle satellite cells were transfected with 0.5 μg of pcDNA3.1(+)-pAkirin2 or 50 nM of Akirin2-siRNA and cultured in proliferation medium for 24 h. (**A**,**C**) The amount of *Akirin2* mRNA against *GAPDH* mRNA was measured by real-time quantitative PCR. (**B**,**D**) The Akirin2 protein expression was measured by Western blotting. Equal loading was monitored with anti-GAPDH antibody. Data were presented as means ± SE (n = 3). **P < 0.01 and ***P < 0.001 as compared with the control group.

**Figure 4:**
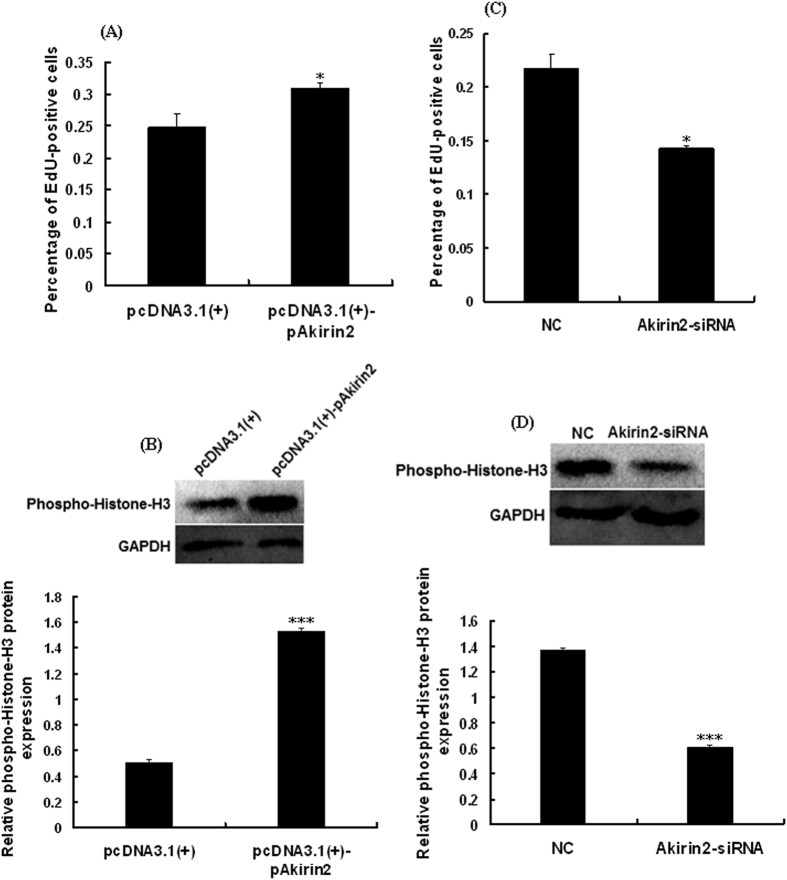
Effect of Akirin2 overexpression and Akirin2 silencing on the proliferation of porcine skeletal muscle satellite cells. Approximately 60% confluent porcine skeletal muscle satellite cells were transfected with 0.5 μg of pcDNA3.1(+)-pAkirin2 or 50 nM of Akirin2-siRNA and cultured in proliferation medium for 24 h. (**A**,**C**) Cell proliferation was evaluated by EdU proliferation assay. The percentage of EdU-positive cells was quantified. Results were presented as mean ± SE (n = 6). (**B**,**D**) Western blot analysis of extracts from porcine skeletal muscle satellite cells by using anti-phospho-Histone H3 antibody. Results were presented as means ± SE (n = 3). *P < 0.05 and ***P < 0.001 as compared with the control group.

Porcine skeletal muscle satellite cells were subjected to cell proliferation analysis after transfection of pcDNA3.1(+)-pAkirin2 plasmid or Akirin2 siRNA for 24 h. The results showed that overexpression of Akirin2 could promote the mRNA (Fig. 3A) and protein expressions of Akirin2 (Fig. 3B) and the cell proliferation (Fig. 4A and B), whereas silencing of Akirin2 inhibited the expression of Akirin2 (Fig. 3C and D) and the cell proliferation (Fig. 4C and D). Taken together, these findings show that Akirin2 functions in promoting the proliferation of porcine skeletal muscle satellite cells.

### Akirin2 promotes differentiation of porcine skeletal muscle satellite cells

**Figure 5:**
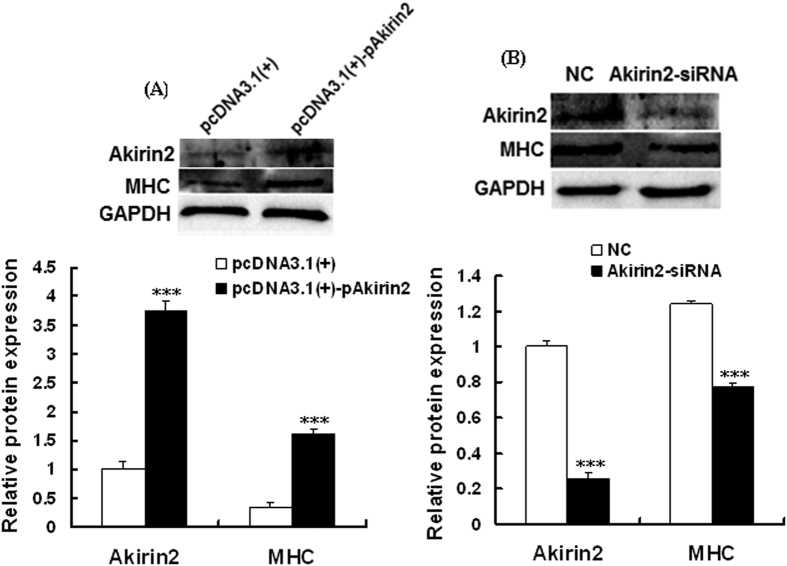
Effect of Akirin2 overexpression and Akirin2 silencing on the differentiation of porcine skeletal muscle satellite cells. (**A**) Overexpression of Akirin2 promoted porcine skeletal muscle satellite cells differentiation. Approximately 80% confluent porcine skeletal muscle satellite cells were transfected with 1 μg of pcDNA3.1(+)-pAkirin2 and cultured in differentiation medium for 72 h. Total cell lysates were subjected to SDS-PAGE and immunoblotted with anti-Akirin2, MHC and GAPDH antibodies. (**B**) Akirin2 silencing inhibited porcine skeletal muscle satellite cells differentiation. Approximately 80% confluent porcine skeletal muscle satellite cells were transfected with 50 nM of Akirin2-siRNA and cultured in differentiation medium for 72 h. Total cell lysates were subjected to SDS-PAGE and immunoblotted with anti-Akirin2, MHC and GAPDH antibodies. Results were presented as means ± SE (n = 3). ***P < 0.001 as compared with the control group.

To investigate the role of Akirin2 in differentiation of porcine skeletal muscle satellite cells, we performed overexpression or silencing of Akirin2 in differentiating cells and measured the protein level of myogenic marker MHC. As shown in Fig. 5, overexpression of Akirin2 significantly increased, whereas silencing of Akirin2 significantly decreased, the protein level of MHC, suggesting that Akirin2 functions in promoting the differentiation of porcine skeletal muscle satellite cells.

### Involvement of ERK1/2 signaling pathway in Akirin2-induced proliferation and differentiation of porcine skeletal muscle satellite cells

**Figure 6:**
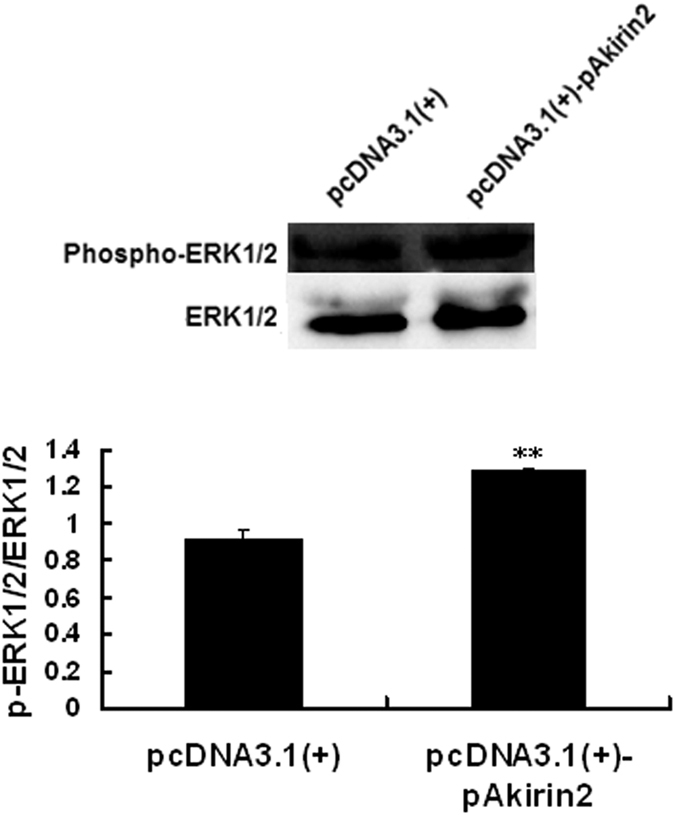
Akirin2 activates ERK1/2 signaling pathway in proliferating porcine skeletal muscle satellite cells. Approximately 60% confluent porcine skeletal muscle satellite cells were transfected with 0.5 μg of pcDNA3.1(+)-pAkirin2 and cultured in proliferation medium for 24 h. Total cell lysates were subjected to SDS-PAGE and immunoblotted with anti-phospho-ERK1/2 and ERK1/2 antibodies. Results were presented as mean ± SE (n = 3). ***P < 0.001 as compared with the control group.

**Figure 7:**
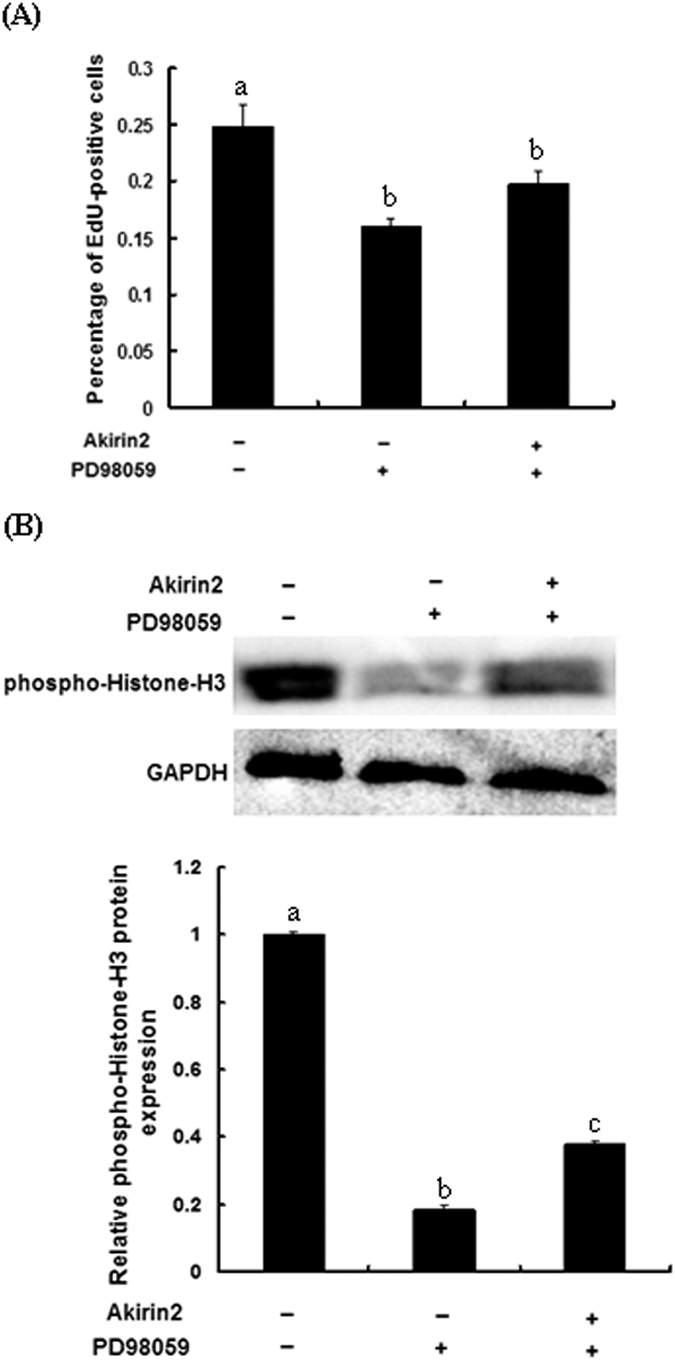
Akirin2 promotes porcine skeletal muscle satellite cells proliferation through ERK1/2 signaling pathway. (**A**) Porcine skeletal muscle satellite cells were cultured in proliferation medium. PD98059 (50 μM) or DMSO was added 1 h before transfection with 0.5 μg of pcDNA3.1(+)-pAkirin2 in approximately 60% confluent porcine skeletal muscle satellite cells. After 24 h, cell proliferation was evaluated by EdU proliferation assay. The percentage of EdU-positive cells was quantified. Results were presented as mean ± SE (n = 6). (**B**) Lysates from porcine skeletal muscle satellite cells treated as described in (**A**) were immunoblotted with anti-phospho-Histone H3 antibody. Equal loading was monitored with anti-GAPDH antibody. Results were presented as mean ± SE (n = 3). Values with different letters are significantly different (P < 0.05).

**Figure 8:**
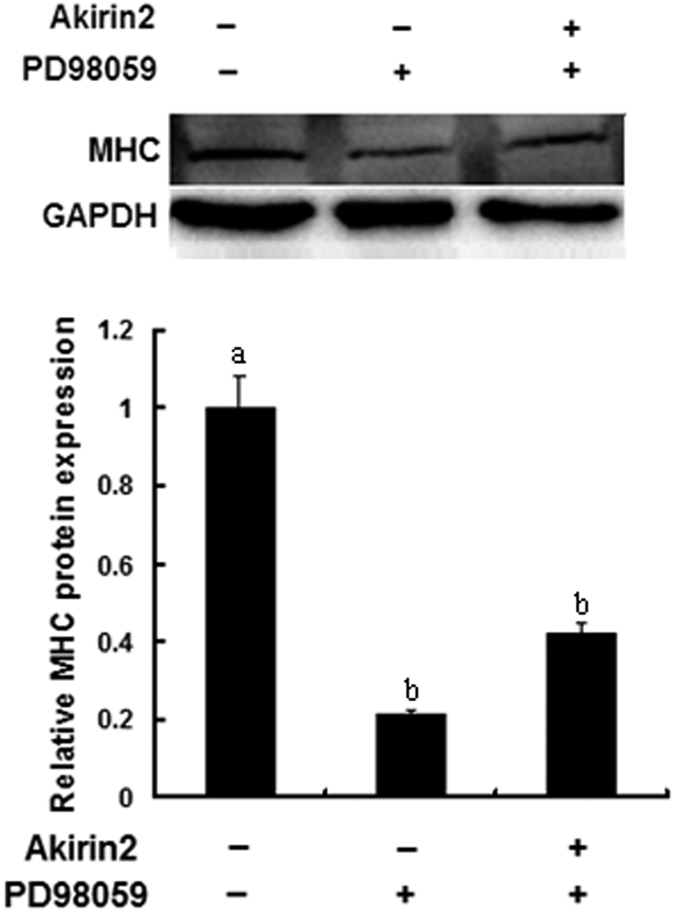
Akirin2 promotes porcine skeletal muscle satellite cells differentiation through ERK1/2 signaling pathway. PD98059 or DMSO was added 1 h before transfection with 1 μg of pcDNA3.1(+)-pAkirin2 in approximately 80% confluent porcine skeletal muscle satellite cells. On day 3 of differentiation, total cell lysates were subjected to SDS-PAGE and immunoblotted with anti-MHC and GAPDH antibodies. Results were presented as mean ± SE (n = 3). Values with different letters are significantly different (P < 0.05).

To investigate whether Akirin2 affects the ERK1/2 signaling pathway, we probed for phospho-ERK1/2 levels in lysates from Akirin2-overexpressed porcine skeletal muscle satellite cells. The results indicated that overexpression of Akirin2 activated ERK1/2 in proliferating cells (Fig. 6). A similar result was also observed in differentiating cells (data not shown). To determine whether Akirin2 affects proliferation and differentiation of porcine skeletal muscle satellite cells through the ERK1/2 signaling pathway, porcine skeletal muscle satellite cells were treated with specific ERK inhibitor PD98059 and Akirin2 overexpression. The results indicated that inhibition of ERK1/2 signaling pathway significantly eliminated the proliferation (Figs 4A,B and 7) and differentiation (Figs 5A and 8) promotion by Akirin2 overexpression.

### Involvement of NFATc1 signaling pathway in Akirin2-induced proliferation and differentiation of porcine skeletal muscle satellite cells

**Figure 9:**
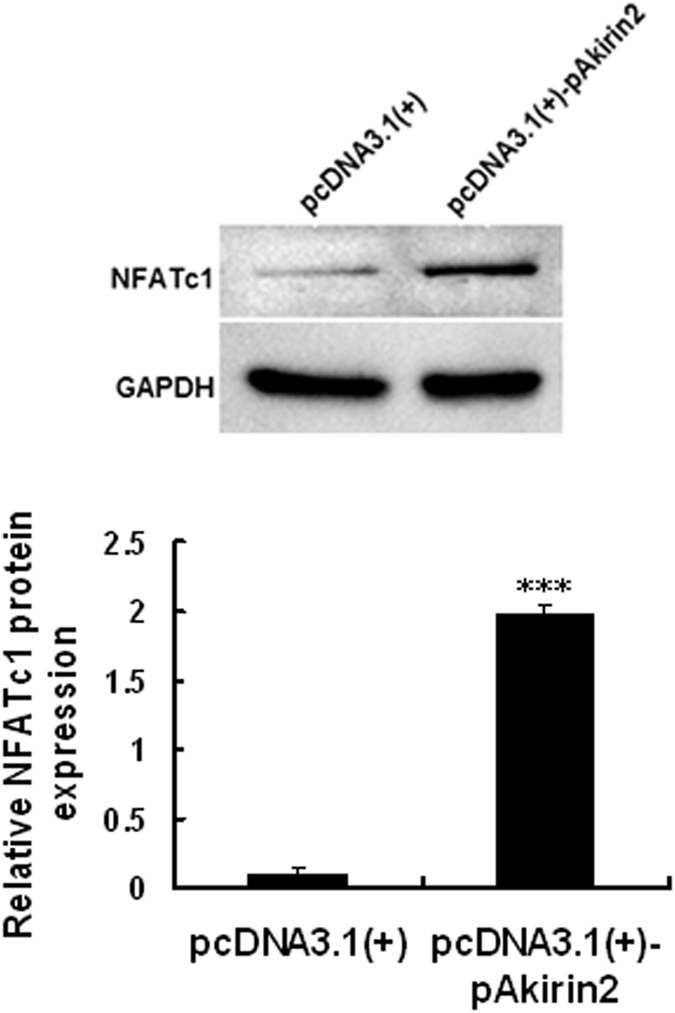
Akirin2 activates NFATc1 signaling pathway in proliferating porcine skeletal muscle satellite cells. Total cell lysates prepared as described in Fig. 6 were subjected to SDS-PAGE and immunoblotted with anti-NFATc1 and GAPDH antibodies. Results were presented as mean ± SE (n = 3). ***P < 0.001 as compared with the control group.

**Figure 10:**
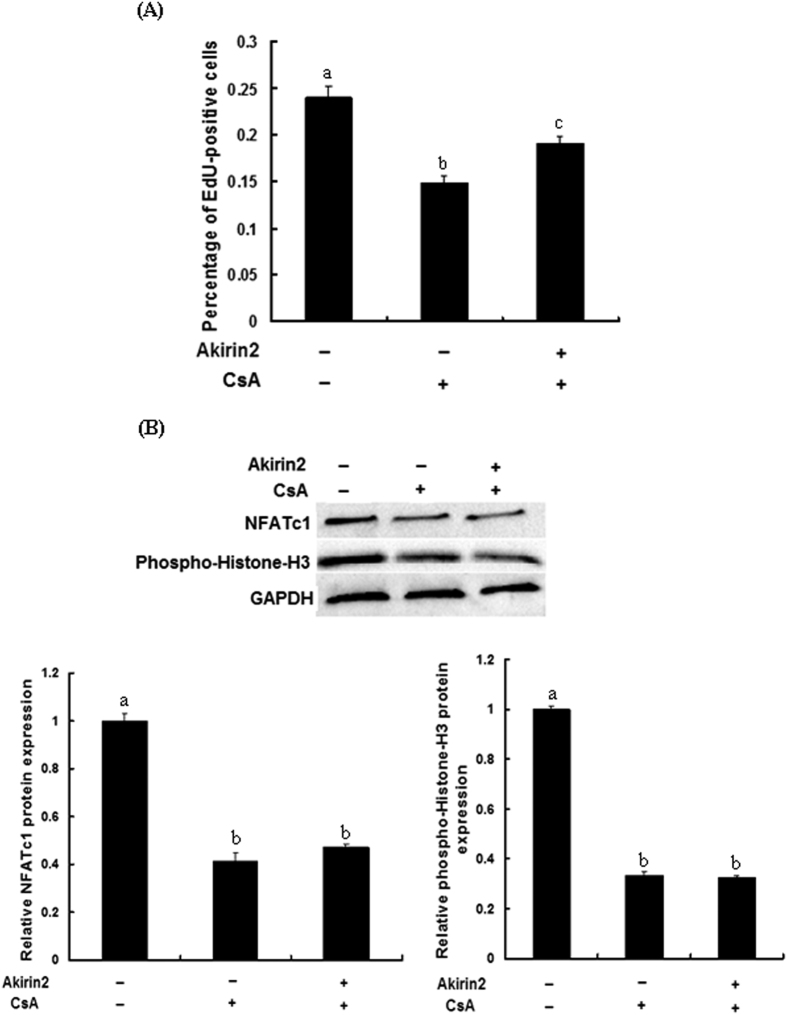
Akirin2 promotes porcine skeletal muscle satellite cells proliferation through NFATc1 signaling pathway. (**A**) Approximately 60% confluent porcine skeletal muscle satellite cells, pretreated with 5 μM CsA for 1 h or left untreated, were transfected with 0.5 μg of pcDNA3.1(+)-pAkirin2. After 24 h, cell proliferation was evaluated by EdU proliferation assay. The percentage of EdU-positive cells was quantified. Data were presented as mean ± SE (n = 6). (**B**) Lysates from porcine skeletal muscle satellite cells treated as described in (**A**) were immunoblotted with anti-phospho-Histone H3 and NFATc1 antibodies. Equal loading was monitored with anti-GAPDH antibody. Results were presented as mean ± SE (n = 3). Values with different letters are significantly different (P < 0.05).

**Figure 11:**
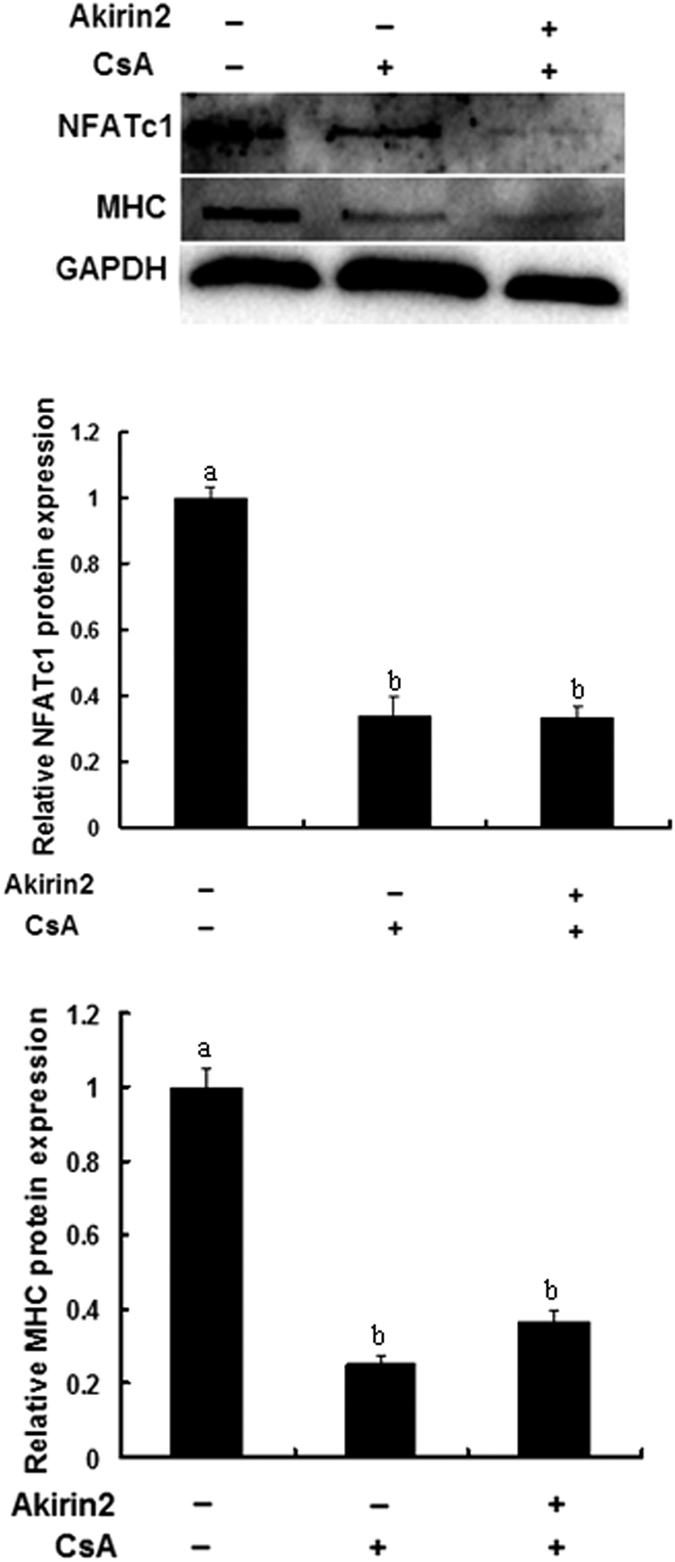
Akirin2 promotes porcine skeletal muscle satellite cells differentiation through NFATc1 signaling pathway. CsA or DMSO was added 1 h before transfection with 1 μg of pcDNA3.1(+)-pAkirin2 in approximately 80% confluent porcine skeletal muscle satellite cells. On day 3 of differentiation, total cell lysates were subjected to SDS-PAGE and immunoblotted with anti-NFATc1, MHC and GAPDH antibodies. Data were presented as mean ± SE (n = 3). Values with different letters are significantly different (P < 0.05).

To investigate whether Akirin2 affects the NFATc1 signaling pathway, we probed for NFATc1 levels in lysates from Akirin2-overexpressed porcine skeletal muscle satellite cells. The results showed that overexpression of Akirin2 increased the protein expression of NFATc1 in proliferating cells (Fig. 9). A similar result was also observed in differentiating cells (data not shown). To verify whether NFATc1 signaling pathway is involved in Akirin2-induced proliferation and differentiation promotion of porcine skeletal muscle satellite cells, the cells were treated with the inhibitor CsA and Akirin2 overexpression. The results showed that the inhibitor CsA significantly eliminated the proliferation (Figs 4A,B and 10) and differentiation (Figs 5A and 11) promotion by Akirin2 overexpression.

### Interaction effects between ERK1/2 and NFATc1 signaling pathways in porcine skeletal muscle satellite cells

**Figure 12:**
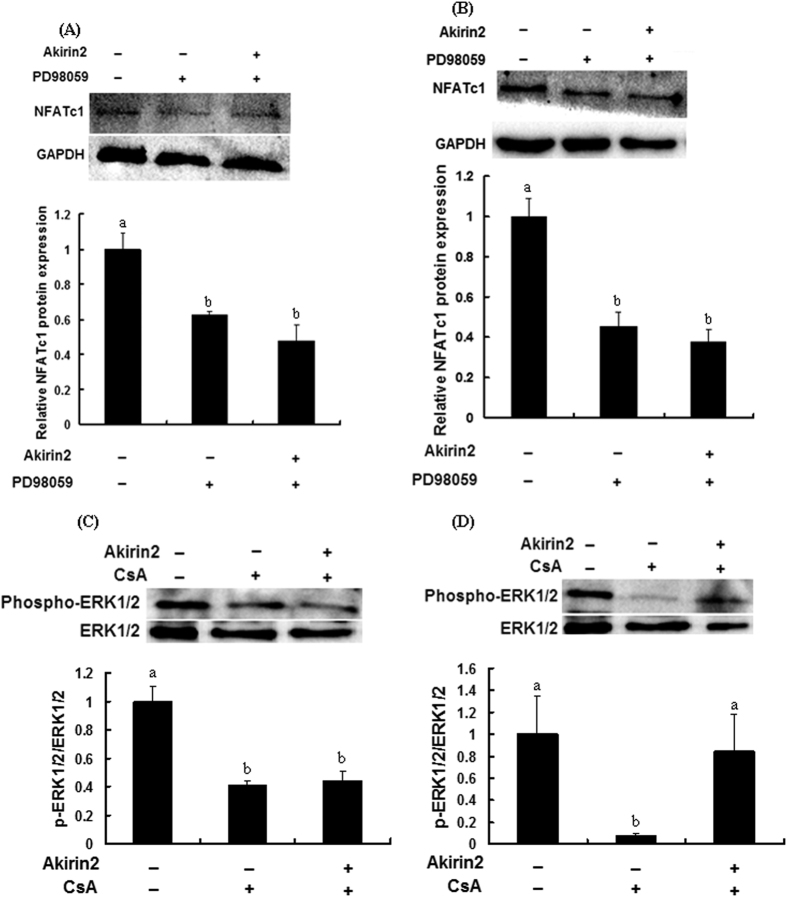
ERK1/2 and NFATc1 signaling pathways interacted with each other in porcine skeletal muscle satellite cells. (**A**,**B**) PD98059 or DMSO was added 1 h before the transfection of pcDNA3.1(+)-pAkirin2. After cell proliferation (**A**) treatment with Akirin2 for 24 h or differentiation (**B**) treatment with Akirin2 for 72 h, total cell lysates were subjected to SDS-PAGE and immunoblotted with anti-NFATc1 and GAPDH antibodies. (**C**,**D**) CsA or DMSO was added 1 h before the transfection of pcDNA3.1(+)-pAkirin2. After cell proliferation (**C**) treatment with Akirin2 for 24 h or differentiation (**D**) treatment with Akirin2 for 72 h, total cell lysates were subjected to SDS-PAGE and immunoblotted with anti-phosphorylation of ERK1/2 and ERK1/2 antibodies. Results were presented as mean ± SE (n = 3). Values with different letters are significantly different (P < 0.05).

Porcine skeletal muscle satellite cells were treated with inhibitors PD98059 or CsA for 1 h before transfection with pcDNA3.1(+)-Akirin2 plasmid for 24 h in proliferation medium and for 72 h in differentiation medium, respectively. The results of Western blot indicated that PD98059 and CsA inhibited NFATc1 and phosphorylation of ERK1/2, respectively, in both proliferation and differentiation medium (Fig. 12).

## Discussion

The process of myogenesis is controlled by several myogenic regulatory factors (MyoG, MyoD, Myf5, Myf6, and so on) which further regulate the expression of many muscle specific genes^21^. This process is also guided by various environmental cues^3^,^4^. There is some evidence that many peptidic factors are able to regulate skeletal muscle cells proliferation or differentiation through distinct signaling pathways^9^,^22^. Akirin2 has been identified as a potential regulator of myogenesis^15^. Here, we addressed the questions whether Akirin2 might participate in regulation of skeletal muscle satellite cells proliferation and differentiation. The results of our work demonstrated that Akirin2 promotes proliferation and differentiation of porcine skeletal muscle satellite cells.

Phospho-Histone H3 protein was used as a mitotic cell cycle biomarker for cell proliferation^23^. MHC is the late differentiation markers of myoblasts^24^,^25^. To study the effects of Akirin2 on the proliferation and differentiation of porcine skeletal muscle satellite cells, we used two approaches: over-expression and RNA interference with Akirin2. Our results showed that overexpression of Akirin2 significantly increased the abundance of phospho-Histone-H3, a S/G2 and G2/M phase marker protein. Furthermore, overexpression of Akirin2 significantly increased the protein expression levels of MHC. Moreover, silencing of Akirin2 decreased the proliferation and differentiation of porcine skeletal muscle satellite cells. Our *in vitro* study revealed that Akirin2 plays an important role in proliferation and differentiation of porcine skeletal muscle satellite cells. However, Sun *et al*. reported that Akirin2 could promote the proliferation but not the differentiation of duck myoblasts^26^. Those findings suggest that the function of Akirin2 in skeletal myogenesis exists the cells or species difference.

There is a lot of evidence to indicate that ERK1/2 pathway is involved in regulating the proliferation of muscle cells^27^,^28^,^29^. To gain insight into the mechanisms by which Akirin2 stimulates the proliferation and differentiation of porcine skeletal muscle satellite cells, we evaluated the signaling events. We found that Akirin2 increased the phosphorylation level of ERK1/2 in proliferating porcine skeletal muscle satellite cells. To test the functional role of ERK1/2 activation induced by Akirin2 in porcine skeletal muscle satellite cells proliferation, we next explored the effects of ERK1/2 inhibitor (PD98059) on Akirin2-induced proliferation promotion of porcine skeletal muscle satellite cells. Inhibition of the ERK1/2 pathway by PD98059 decreased Akirin2-induced proliferation promotion of porcine skeletal muscle satellite cells. These results suggest that the ERK1/2 pathway mediates the stimulatory effects of Akirin2 on the proliferation of porcine skeletal muscle satellite cells.

We have also demonstrated that Akirin2 promotes the myogenic differentiation of porcine skeletal muscle satellite cells. The stimulatory effect was characterized by increasing protein expression level of MHC, a myogenic differentiation-related protein. Previous studies suggested that the effect of MAPK on muscle cells differentiation is controversial. Some studies suggest that activation of the ERK pathway prevents skeletal muscle differentiation^29^,^30^,^31^, and other studies believe that it functions at two stages of skeletal muscle differentiation^10^,^32^. However, recent data indicate that ERK1/2 may positively regulate myogenic differentiation^6^,^28^,^33^. In this study, we showed that Akirin2 promotes differentiation of porcine skeletal muscle satellite cells through ERK1/2 signaling pathway.

Calcineurin (CaN) has been reported to be a possible candidate in the signaling of skeletal muscle cellular growth, and plays an important role in regulating cell proliferation^34^ and differentiation^35^,^36^,^37^. CaN has also been reported to affect the muscle regeneration by association with NFATc1, a downstream target of CaN signaling^37^. As Akirin2 affects both the proliferation and differentiation of porcine skeletal muscle satellite cells, we hypothesized that Akirin2 affects the proliferation and differentiation of porcine skeletal muscle satellite cells via the NFATc1 signaling pathway. The immunosuppressive drug CsA is a well-known inhibitor of CaN, and thus inhibits NFAT activity by blocking its dephosphorylation^38^. CsA has also been reported to inhibit myoblast differentiation^35^,^36^. Here we demonstrated that CsA inhibited proliferation and differentiation of porcine skeletal muscle satellite cells, and Akirin2 promoted the NFATc1 protein expression in porcine skeletal muscle satellite cells during both proliferation and differentiation stages.

In the present study, we demonstrated that Akirin2 activated both ERK1/2 and NFATc1 signaling pathway in porcine skeletal muscle satellite cells. So we speculated that ERK1/2 and NFATc1 signaling pathways might crosstalk with each other. This speculation was partly supported by the data of the present study. Further study is warranted to verify the speculation more thoroughly.

In summary, the present study demonstrated that Akirin2 plays an important role in proliferation and differentiation of porcine skeletal muscle satellite cells, and further revealed that Akirin2 promotes proliferation and differentiation of porcine skeletal muscle satellite cells through ERK1/2 and NFATc1 signaling pathways. However, it is necessary to further investigation of the role of Akirin2 in skeletal muscle development by *in vivo* study.

## Materials and Methods

### Ethics statement

This study was carried out in strict accordance with the recommendations in the Guide for the Care and Use of Laboratory Animals of Sichuan Agricultural University. All experimental protocols were approved by the Animal Care Advisory Committee of Sichuan Agricultural University.

### Reagents

The calcineurin inhibitor cyclosporin A (CsA) was obtained from Amresco (USA) and resolved in DMSO. The specific ERK inhibitor PD98059 was purchased from Sigma (St. Louis, MO, USA) and resolved in DMSO, then used at 50 μM. Anti-NFATc1 (Cat. No. 8032) antibody was obtained from Cell signaling Technology (Danvers, MA, USA). Anti-phospho-ERK1/2 (Cat. No. sc-16982), phospho-Histone H3 (Cat. No. sc-8656-R), MHC (Cat. No. sc-20641) and GAPDH (Cat. No. sc-20357) antibodies were all obtained from Santa Cruz Biotechnology (Santa Cruz, CA, USA). Anti-ERK1/2 (Cat. No. 16443–1-AP) antibody was obtained from ProteinTech Biotechnology (Chicago, IL, USA).

### Isolation of porcine skeletal muscle satellite cells

Porcine skeletal muscle satellite cells were isolated from 3-day-old male Duroc × Yorkshire × Landrace (DLY) pigs as described previously^39^ with some modifications. Briefly, skeletal muscles were digested with 0.2% collagenase type II (Sigma) and then filtered successively through 200-mesh and 400-mesh cell sieves. The collected cells were purified by differential adhesion method. The resulting mononuclear cells were cultured in DMEM/F12 (Invitrogen) supplemented with 20% FBS, 100 U/mL penicillin and 100 μg/μL streptomycin at 37 °C in a humidified 5% CO_2_ atmosphere. The cells were identified by immunofluorescence with anti-Pax7 antibody (PAX7, DSHB, USA) (data not shown).

### Cell culture

For stimulation experiments, cells were treated with recombinant plasmid pcDNA3.1(+)-pAkirin2, and PD98059 (ERK1/2 inhibitor) or cyclosporin (CsA, CaN inhibitor) were added 1 h before the treatment. Myogenic differentiation was induced by changing the medium to DMEM/F12 supplemented with 2% horse serum and penicillin/streptomycin.

### The siRNA and plasmid transfection

A pair of 21-nucleotide siRNA sequences targeting Akirin2 was designed and synthesized by GenePharm (Shanghai, China). The sense strand of the Akirin2 siRNA was 5′-GCUGUACUUCUGAUGCACATT-3′, and the antisense strand was 5′-UGUGCAUCAGAAGUACAGCTT-3′. The sense strand of the negative control siRNA was 5′-UUCUCCGAACGUGUCACGUTT-3′, and the antisense strand was 5′-ACGUGACACGUUCGGAGAATT-3′. The siRNA was dissolved in DEPC-treated water, and the final concentration was 50 nM. The pcDNA3.1(+)-pAkirin2 plasmid was constructed by our lab^20^. The lipofectamine 2000 (Invitrogen, California, USA) was used to transfect the porcine skeletal muscle satellite cells according to the manufacturer’s instruction.

### Cell proliferation analysis

To investigate cell proliferation, markers of two phases of the cell cycle were analyzed by using EdU (5-ethynyl-2′-deoxyuridine) for synthesis phase and phospho-histone H3 for G2-M phase. EdU proliferation assay was performed as described by Chen *et al*.^40^ using a Click-iT EdU Alexa Fluor 594 Imaging Kit (Invitrogen). The level of phospho-histone H3 protein was detected by western blot analysis.

### RNA extraction and real-time quantitative PCR

Total RNA isolation was performed according to the RNAiso Plus reagent (TaKaRa, Dalian, China) protocol. cDNA was synthesized by using PrimeScript^®^ RT reagent Kit with gDNA Eraser (TaKaRa) according to the manufacture’s instructions. For real-time quantitative PCR analysis, synthesized cDNA and SYBR select Master Mix (Applied Biosystems, Foster, CA, USA) were run on an ABI 7900HT Real-time PCR system. The gene specific primers used are listed in Table 1. The PCR cycling conditions were as following: 45 cycles at 95 °C for 15 s and 60 °C for 30 s. Relative gene expression was determined using the comparative Ct method^41^ with *GAPDH* as an endogenous control.

### Western blotting

Protein was extracted from porcine skeletal muscle satellite cells using RIPA cell lysis buffer (Pierce, Rockford, IL, USA) supplemented with protease inhibitor cocktail (Sigma). Protein concentrations were assessed by BCA protein assay kit (Pierce). Equal amounts of protein were loaded onto 10% sodium dodecyl sulfate-polyacrylamide gel and transferred to a nitrocellulose membrane. The membrane was blocked in 3% non-fat milk in TBS-0.1% Tween-20 for 1 h and incubated overnight with primary antibody at 4 °C, followed by horseradish peroxidase-linked secondary antibodies (Santa Cruz Biotechnology) for 1 h at 37 °C. The bound antibodies were visualized with a Clarity^TM^ Western ECL Substrate (Bio-Rad, Hercules, CA, USA) using a ChemiDoc XRS Imager System (Bio-Rad). Housekeeping protein GAPDH was used as a control for equal protein loading. The density of the protein bands was determined using Gel-Pro Analyzer 4.2 software (Media Cybernetics, Rockville, MD, USA).

### Statistical analysis

All data, expressed as mean ± SE, were subjected to one-way ANOVA analysis or Tukey test using SPSS 11.0 software and P < 0.05 was considered significant.

## Additional Information

**How to cite this article:** Chen, X. *et al*. Akirin2 regulates proliferation and differentiation of porcine skeletal muscle satellite cells via ERK1/2 and NFATc1 signaling pathways. *Sci. Rep.* **7**, 45156; doi: 10.1038/srep45156 (2017).

**Publisher's note:** Springer Nature remains neutral with regard to jurisdictional claims in published maps and institutional affiliations.

## Figures and Tables

**Table 1:** List of genes, primer sequences, GenBank accession numbers, and product sizes.

Gene name	Primer	Sequence	GenBank accession no.	Product size (bp)
*Akirin2*	Forward	5′-GCATTTCTCCTCAGTGGAC-3′	**JN227885**	93
	Reverse	5′-CTGCCGTAGGGTGAATAAG-3′		
*Myogenin*	Forward	5′-CGCCATCCAGTACATCGAG-3′	**NM_001012406**	125
	Reverse	5′-TGTGGGAACTGCATTCACTG-3′		
*GAPDH*	Forward	5′-ACACTGAGGACCAGGTTGTG-3′	**NM_001206359**	98
	Reverse	5′-GACGAAGTGGTCGTTGAGGG-3′		
